# Activating SIRT1 deacetylates NF-κB p65 to alleviate liver inflammation and fibrosis via inhibiting NLRP3 pathway in macrophages

**DOI:** 10.7150/ijms.77955

**Published:** 2023-02-21

**Authors:** Shuli He, Yaru Wang, Jinying Liu, Pengju Li, Xiaoying Luo, Bingyong Zhang

**Affiliations:** 1 Department of Gastroenterology, Zhengzhou University People's Hospital, Henan Provincial People's Hospital, Henan University People's Hospital, Zhengzhou, 450003, China; 2 Microbiome Laboratory, Zhengzhou University People's Hospital, Henan Provincial People's Hospital, Henan University People's Hospital, Zhengzhou, 450003, China

**Keywords:** liver fibrosis, macrophages, SIRT1, NF-κB p65

## Abstract

**Background and aims:** Macrophages play a critical role in the development of liver diseases. As an NAD^+^-dependent histone deacetylase, SIRT1 inhibits liver inflammation and fibrosis, but the mechanisms are not fully understood. Our aim was to investigate the molecular mechanism of SIRT1 in macrophages in liver inflammation and fibrosis.

**Methods:** We employed the CCl4-induced hepatic fibrosis rat models and cultured

murine macrophages RAW 264.7 *in vitro* to explore the anti-fibrosis effect of SIRT1. The content of cytokines was measured with ELISA. The expression of proteins associated with the NF-κB /NLPR3 signaling pathway was detected by Western blot, co-immunoprecipitation, and immunofluorescence. SIRT1, NF-κB, and NLRP3 genes were knocked down in RAW 264.7 cells by small interfering RNA (siRNA) transfection.

**Results:** The expression of NF-κB p65, NLRP3, α-SMA, and iNOS increased in liver tissue, with high plasma LPS level and low expression of SIRT1 in CCl4-induced rat models. Overexpressing SIRT1 could inhibit these protein levels, decrease plasma LPS level, and attenuate liver injury and fibrosis. *In vitro*, LPS induced cytomorphology changes and up-regulated NF-κB/NLRP3 pathway, with the low expression of SIRT1 in RAW 264.7; meanwhile, the secretion of inflammatory factors increased. Nevertheless, knockdown of NF-κB or NLRP3 and activation of SIRT1 inhibited inflammation of macrophages; inhibition or knockdown of SIRT1 enhanced macrophage inflammation. Furthermore, activation of SIRT1 could inhibit LPS-treated macrophages from activating hepatic stellate cells (HSCs).

**Conclusions:** Activating SIRT1 inhibits the inflammation in macrophages by down-regulating NLRP3 pathway through deacetylating NF-κB p65, which in turn inhibits the activation of HSCs to alleviate hepatic inflammation and fibrosis.

## Introduction

Hepatic fibrosis is an excessive wound-healing response to persistent liver injury in a variety of chronic liver diseases, which can further develop into cirrhosis and hepatocellular carcinoma 1. Although the pathophysiology of liver fibrosis is multifactorial, liver macrophages, which are mainly composed of resident macrophages (called KCs) and monocyte-derived macrophages (MoMFs), have become the core participants in the development and regression of liver fibrosis 2, 3. Furthermore, activation of macrophages mediates local inflammation, and the transcription factor NF-κB signaling pathway is a key mediator of the macrophage immune response 4. The research indicates that the activation of macrophages is closely associated with liver inflammation and fibrosis. Thus, it is crucial to understand the role of macrophages in hepatic inflammation and fibrosis.

NLRP3 inflammasome, a cytoplasmic complex, contains NLRP3, the caspase recruitment domain (ASC), and caspase-1, and is an important regulator of the innate immune system 5. When stimulated, NLRP3 binds with its adaptor ASC and catalyzes pro-caspase-1 to form active caspase-1, thereby promoting the secretion of inflammatory factors 6. This step is a prerequisite for releasing cytokines, which in turn have pro-inflammatory effects on surrounding cells 7. Since NLRP3 inflammasome is widely recognized as one of the core regulators of liver injury and inflammation 8, we hypothesized that NLRP3 may lead to liver inflammation and fibrosis by activating macrophages.

SIRT1, a class Ⅲ histone deacetylase, is an essential regulator of metabolism, cell survival, and longevity 9. Resveratrol, a potent SIRT1 activator, is a polyphenol plant antitoxin that significantly reduces the production of pro-inflammatory mediators and cytokines 10. SIRT1 is a key immunomodulator by suppressing inflammation or modulating immune cell activation 11. For example, when the lungs are exposed or animals are injected with LPS, SIRT1-deficient macrophages showed higher levels of tumor necrosis factor-α (TNF-α) and Interleukin-1β (IL-1β) 12. Besides, recent studies emphasize that activating SIRT1 ameliorates liver fibrosis by inhibiting hepatic inflammation 13, 14. However, the mechanisms by which SIRT1 reduces liver inflammation and fibrosis are still not fully understood.

Accordingly, abnormal activation of macrophages and hepatic stellate cells was observed in liver tissue of CCl4-induced rat liver fibrosis models. Furthermore, we investigated the effect of SIRT1 on macrophages during liver inflammation and fibrosis *in vitro*, and explored possible mechanisms. The results may provide a novel insight into the treatment of liver fibrosis.

## Materials and methods

### Cell lines and reagents

Cells included murine macrophages RAW 264.7 (337875) (BNCC, Beijing, China) and primary murine HSCs (isolated from male SD rats). Reagents included carbon tetrachloride (56235) (Sigma-Aldrich, Shanghai, China), Lipopolysaccharide (LPS; S1732) (Beyotime, Hainan, China), resveratrol (SC0276) (Beyotime, Hainan, China), selisistat (49843-98-3) (EX-527; MedChemExpress, NJ, USA), DAPI (D9542) (Sigma-Aldrich, Shanghai, China), Alexa Fluor™ 647 Phalloidin (A22287) (Thermo, Shanghai, China), protease cocktails inhibitor (P1005) (Beyotime, Hainan, China) and Phenylmethanesulphonyl fluoride (ST506) (PMSF; Beyotime, Hainan, China).

### Animal experimental design

Sprague-Dawley (SD) rats were provided by the Experimental Animal Center (Henan University of Traditional Chinese Medicine, China) (the certificate number is 41003100006844) and were approved by the Animal Experiment Ethics Committee of Southern Medical University (Ethics Approval Code: 1912033). Animals were fed at 22 -24°C with light/darkness cycles of 12:12 h.

#### Establishment of the rat model of liver fibrosis induced by carbon tetrachloride

Normal male SD rats (180-220 g, 6 weeks of age) were subjected to intraperitoneal injection with 40% carbon tetrachloride (CCl4) -olive oil solution at 2 ml /kg body weight twice a week for 28 days, CCl4-induced rat models were randomly sacrificed on days 6 and 28 (n = 6 for each group).

#### The treatment of SIRT1 adenovirus vector

To investigate the role of SIRT1 in liver inflammation and fibrosis, we used the GFP-SIRT1-adenovirus vector and the GFP-blank vector produced by Hanbio AdenoVector Institute (Shanghai, China). One week before intraperitoneal injection of CCl4-olive oil solution, 10^11^ doses of virus particles were injected through the tail vein to rats. We used the CCl4-induced hepatic fibrosis rat model (n = 6 per group for 6 days and n = 6 per group for 28 days). The vehicle group (n = 6 per group for 6 days and n = 6 per group for 28 days) received intraperitoneal injection of the same volume of olive oil twice a week for 28 days. AV CTR+CCl4 group and AV-SIRT1+CCl4 group (n = 6 per group for 6 days and n = 6 per group for 28 days) were intraperitoneally injected with CCl4-olive oil solution twice a week after administering vectors. On Day 6 and 28, the rats were randomly sacrificed. The SIRT1 sequences were used: sense (5-CGGGCCCTCTAGACTCGAGCGGCCGCATGATTGGCACCGATCCTC-3).

### Histological analysis and immunohistochemistry

Paraffin sections (4μm) of model rat liver tissue were prepared by hematoxylin and eosin (H & E) staining and Masson staining. ISHAK and Metavir score used to assess liver histological inflammation and fibrosis stages in rats. Immunohistochemical detection of SIRT1, NLRP3, NF-κB p65, α-SMA, and iNOS was performed on paraffin sections (3 μm) of liver tissue. The sections were then exposed to HRP-antibody colored with DAB and observed under a microscope (BX51, Olympus, Japan). The number of SIRT1, NF-κB p65, NLRP3, α-SMA, or iNOS positive cells was quantified with Image J software. The first step of immunohistochemical quantitative analysis was to detect gray values in IHC images. Then the gray value was converted to optical density. Staining intensity was measured as “average optical density”. Then the average staining intensities for all measured cells from 5 fields of vision were counted for each sample. Finally, statistical analysis was performed using GraphPad Prism 8.0.1 software.

### Cell experiment design

#### Cell culture and treatment

Murine macrophages RAW 264.7 were cultured in plates with a medium comprising 90% DMEM (Solarbio, Beijing, China) and 10% foetal bovine serum (FBS, Biological Industries, Kibbutz Beit Haemek, Israel). Murine macrophages RAW 264.7 were stimulated by LPS with a concentration gradient of 0, 10, 20, 50, 100 ng/ml for 24 hours, or a time gradient of 0, 6, 12, 24 hours, or pretreated with resveratrol (10 uM) or EX-527 (1 uM). Primary murine HSCs were cultured in plates with a medium comprising 80% DMEM (Solarbio, Beijing, China) and 20% foetal bovine serum (FBS, Biological Industries, Kibbutz Beit Haemek, Israel) and then co-cultured with macrophages.

#### Small interfering RNA (siRNA) transfection assay

According to the manufacturer's instructions, murine macrophages RAW 264.7 were transfected with siRNA to silence NF-κB, NLRP3 or SIRT1. The transfection efficiency was 75%. The following NF-κB siRNA sequences were used: sense (5-GTCAGAGTCTCAAGTATGT-3). The following NLRP3 siRNA sequences were used: sense (5-CAGCCAGAGTGGAATGAGCTA-3). The following SIRT1 siRNA sequences were used: sense (5-CCTCAAGCCATGTTCGATA -3).

#### Cell co-culture

First, murine macrophages RAW 264.7 were planted on 12-well plates. After 40% growth, cells were stimulated with LPS (100 ng/ ml) for 24 h, or pretreated with resveratrol (10 uM). Then the medium was changed and HSCs were planted. After 5 days, the expression of α-SMA and Desmin was detected by immunofluorescence.

#### Western blotting

Murine macrophages RAW 264.7 were treated with various stimulants, then were lysed in lysis buffer containing protease cocktails inhibitor and PMSF, and centrifuged at 12000g, 4℃, for 15 min. Protein concentrations were determined by the BCA method and the equivalent protein supernatants in these groups were isolated by SDS/PAG and transferred to the PVDF membranes. After that, PVDF membranes were sequentially sealed with 5% BSA, and protein levels in murine macrophage RAW 264.7 were determined by western blotting. The primary antibodies included anti-SIRT1 (ab110304) (Abcam, Cambridge, England), anti-NF-κB p65 (bsm-33117M) (Bioss, Beijing, China), anti-acetylated NF-κB p65 Lys310 (Ac NF-κB p65 Lys310) (# 12629) (Cell Signaling Technology, Inc., Boston, MA, USA), anti-NLRP3 (383319) (ZEN, Sichuan, China), anti-caspase-1 (342947) (ZEN, Chengdu, Sichuan), anti-iNOS (381545) (ZEN, Sichuan, China), anti-GAPDH (600041) (Proteintech, Wuhan, China). HRP-conjugated Affinipure Goat Anti-Mouse IgG (511103) (H+L; ZEN, Sichuan, China), and HRP-conjugated Affinipure Goat Anti-Rabbit IgG (511203) (H+L; ZEN, Sichuan, China) were used for secondary antibodies. The protein bands were observed using Pierce™ ECL (Guangdong, China) western blot substrates.

#### Co-immunoprecipitation (Co-IP)

Murine macrophages RAW 264.7 were harvested after being stimulated by 24h with LPS (100 ng/ ml), or pretreated with resveratrol (10uM) before being stimulated by LPS (100ng/ml) and then added to a moderate amount of cell lysis buffer (including protease inhibitor) to extract total protein. The proper amount of lysate was added with corresponding antibodies, and the lysate was slowly shaken and incubated overnight at 4℃. 100ul protein A/ G-sepharose was added to cell lysates incubated with antibodies overnight, and slowly shaken at 4℃ for incubation for 4h, making the antibody conjugate with protein A/G-sepharose. After the immunoprecipitation reaction, centrifugation was performed at 4℃(3000rpm) for 3 minutes, then discarded the supernatant and washed agarose beads 3 times with 1ml lysate. Finally, 15ul of 2×SDS loading buffer was added and boiled for 6min. After cooling, it was placed at -20℃ for subsequent Western blotting. The antibodies for IP included anti-NF-κB p65 and non-specific IgG.

#### Immunofluorescence staining

To reveal NF-κB p65 and iNOS proteins in murine macrophages RAW 264.7 and Desmin and α-SMA in primary murine HSCs, double immunostaining was conducted. Paraformaldehyde-fixed cells were incubated with the primary antibody, followed by the secondary antibody, and then mounted with DAPI. The primary antibodies included anti-NF-κB p65 (1:200), anti-iNOS (1:200), anti-Desmin (16520-1-AP) (Proteintech, Wuhan, China) (1:200), anti-α-SMA (BM0002) (Boster, Wuhan, China) (1:200). The secondary antibodies included FITC-labelled goat anti-rabbit IgG (a0562) (H+L; Beyotime, Hainan, China) (1:200) and Cy3-labelled goat anti-mouse IgG (a0521) (H+L; Beyotime, Hainan, China) (1:200).

### ELISA (Enzyme linked immunosorbent assay)

When the rats were killed, blood samples were collected, serum was collected by centrifugation, and the concentration of LPS in serum was detected by ELISA. When the murine macrophages RAW 264.7 were cultured, the cells were treated with different stimuli, and the supernatants of the cells were collected. The concentrations of IL-1β, TNF-α, and TGF-β (transforming growth factor-β) in the cell supernatants were detected by ELISA.

### Statistical analysis

All values are presented as mean ± SD and were analyzed by analysis of variance (ANOVA) with the statistical software GraphPad Prism 8.0.1. Differences were considered significant at P < 0.05.

## Results

### Overexpressing SIRT1 attenuates CCl4-induced rat liver inflammation

**Figure 1:**
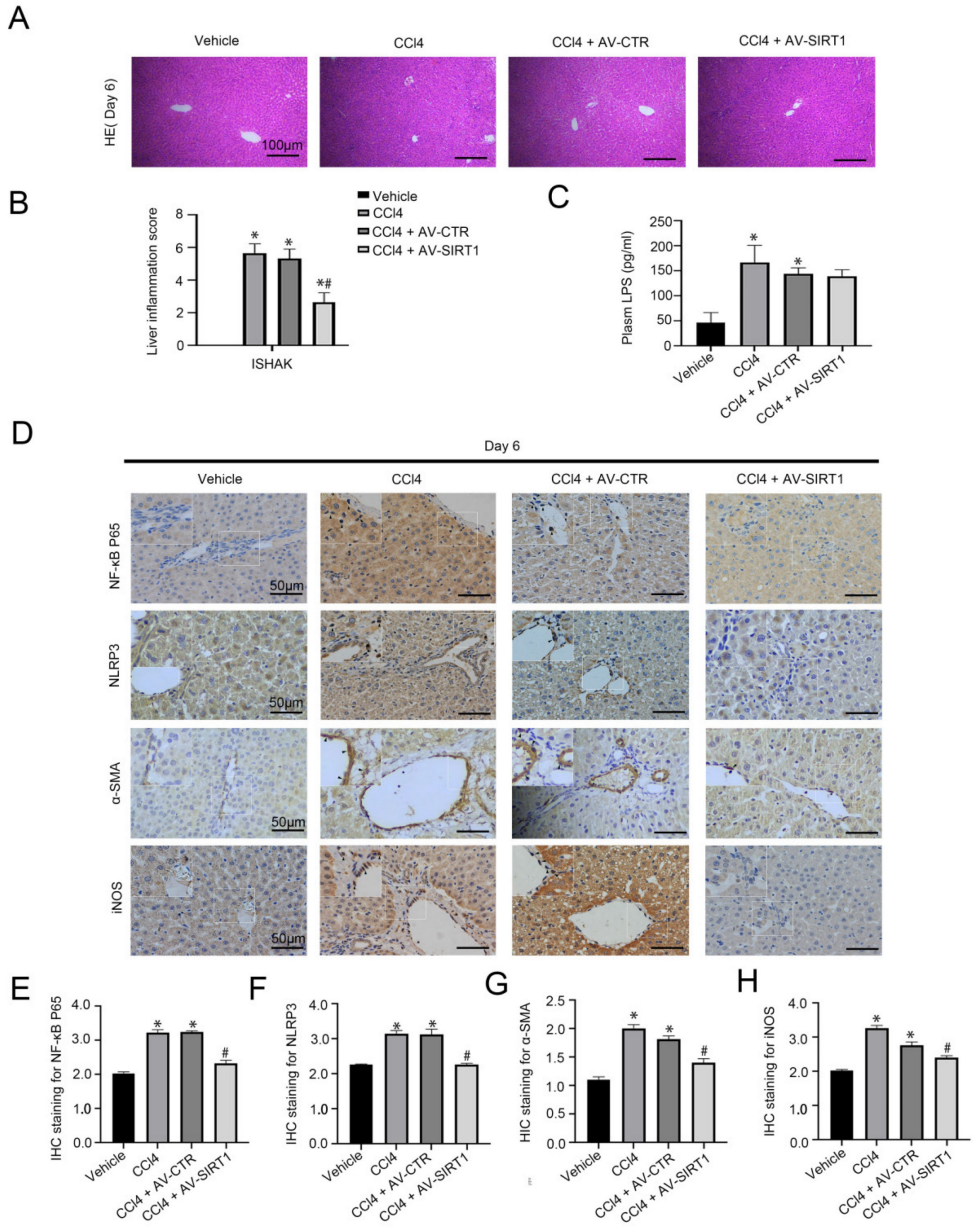
Transfer of SIRT1 gene into CCl4-induced rat model reduces rat liver inflammation. (A) The H&E staining was observed in liver biopsy specimens of CCl4-induced rat models on Day 6 (scale bar: 100 μm). **(**B) The quantified analysis of liver inflammation with ISHAK score. (C) The content of plasma LPS in CCl4-induced rat models on Day 6. (D) The immunohistochemical staining (IHC) for NF-κB P65, NLRP3, α-SMA and iNOS in liver biopsy specimens of CCl4-induced rat models on Day 6 (scale bar: 50 μm). (E-H) The semiquantitative score of IHC staining for NF-κB P65, NLRP3, α-SMA, and iNOS. *P < 0.05 versus the Vehicle group on Day 6; ^#^P < 0.05 versus the CCl4 + AV-CTR group on Day 6.

In our previous research, CCl4 could initiate rat hepatitis on Day 6 15. To evaluate the effect of SIRT1 on macrophages in CCl4-induced acute liver injury, the SIRT1 adenovirus vectors were transferred into CCl4-induced rat models through caudal veins. The H&E staining and the immunohistochemical (IHC) staining showed that compared with the control group, the expression of NF-κB P65, NLRP3, and α-SMA increased in liver tissue of the CCl4 group and the CCl4 + AV-CTR group on Day 6, with high expression of iNOS, which is a pro-inflammatory phenotypic of macrophages; in contrast, overexpressing SIRT1 with adenovirus vector could inhibit these effects (Figure 1A, B, D-H). Additionally, plasma level of LPS was significantly elevated in CCl4-induced rat models on Day 6, which was not inhibited by the SIRT1 adenovirus vector (Figure 1C).

The data suggested that CCl4 triggered circulating LPS to aggravate liver inflammation, which was closely related to the NF-κB/NLRP3 pathway in macrophages. Overexpression of SIRT1 might directly inhibit the NF-κB/NLRP3 pathway and alleviate liver injury in a short duration of time, except for circulating LPS.

### Overexpression of SIRT1 also alleviates CCl4-induced rat liver fibrosis

**Figure 2:**
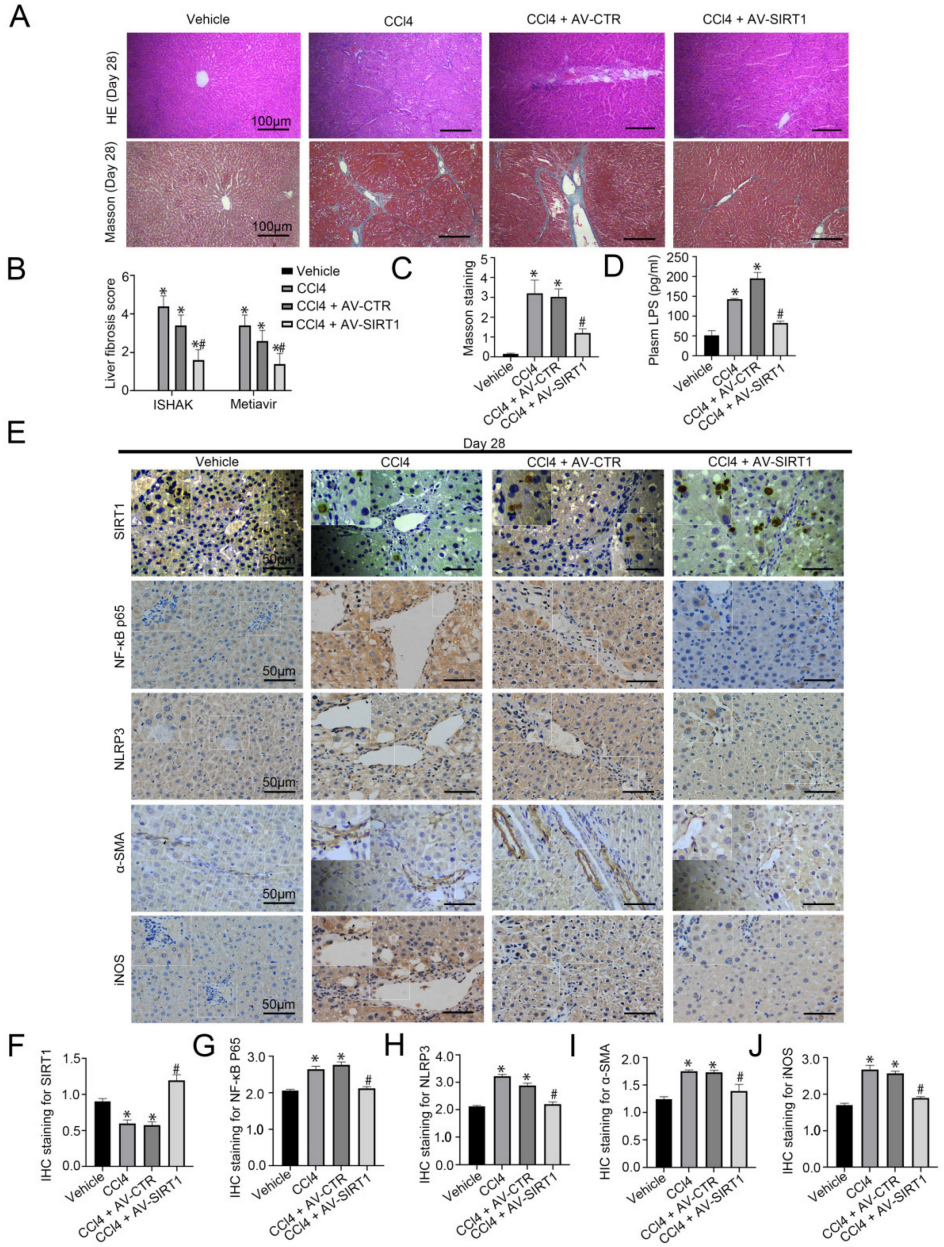
Transfer of SIRT1 gene into CCL4-induced rat model alleviates rat liver fibrosis. (A) The H&E and Masson staining in liver biopsy specimens of CCl4-induced rat models on Day 28 (scale bar: 100 μm). (B) The quantitation with ISHAK and Metavir score in liver biopsy specimens of CCl4-induced rat models on Day 28. (C) The semiquantitative analysis of Masson staining in rat liver tissue on Day 28. (D) Plasma LPS content in CCL4-induced rat models on Day 28. (E) The immunohistochemical staining (IHC) for SIRT1, NF-κB P65, NLRP3, α-SMA and iNOS in liver biopsy specimens of CCl4-induced rat models on Day 28 (scale bar: 50 μm). (F-J) The semiquantitative score of IHC staining for SIRT1, NF-κB P65, NLRP3, α-SMA, and iNOS. *P < 0.05 versus the Vehicle group on Day 28; ^#^P < 0.05 versus the CCl4 + AV-CTR group on Day 28.

Our study has confirmed CCl4-induced rat hepatic fibrosis on Day 28 15. Further, we transferred SIRT1 adenovirus vector to CCl4-induced rat liver fibrosis models. The H&E staining, Masson staining, and the IHC staining showed that overexpression of SIRT1 could relieve liver fibrosis and reduce the expression of NF-κB P65, NLRP3, α-SMA, and iNOS in liver tissue of CCl4-induced liver fibrosis on Day 28 (Figure 2A-C, E-J). Besides, CCl4 triggered the high level of LPS in plasma in rat liver fibrosis, which was inhibited by overexpressing SIRT1 (Figure 2D). These results implied that prolonged CCl4 might elevate circulating LPS to activate inflammation in macrophages and promote liver fibrosis through the up-regulation of NF-κB/NLRP3 pathway, which might be improved by overexpression of SIRT1.

### Macrophages were activated into pro-inflammatory M1 type by LPS and secreted inflammatory cytokines through acetylation of NF-κB p65 and activation of NLRP3

**Figure 3:**
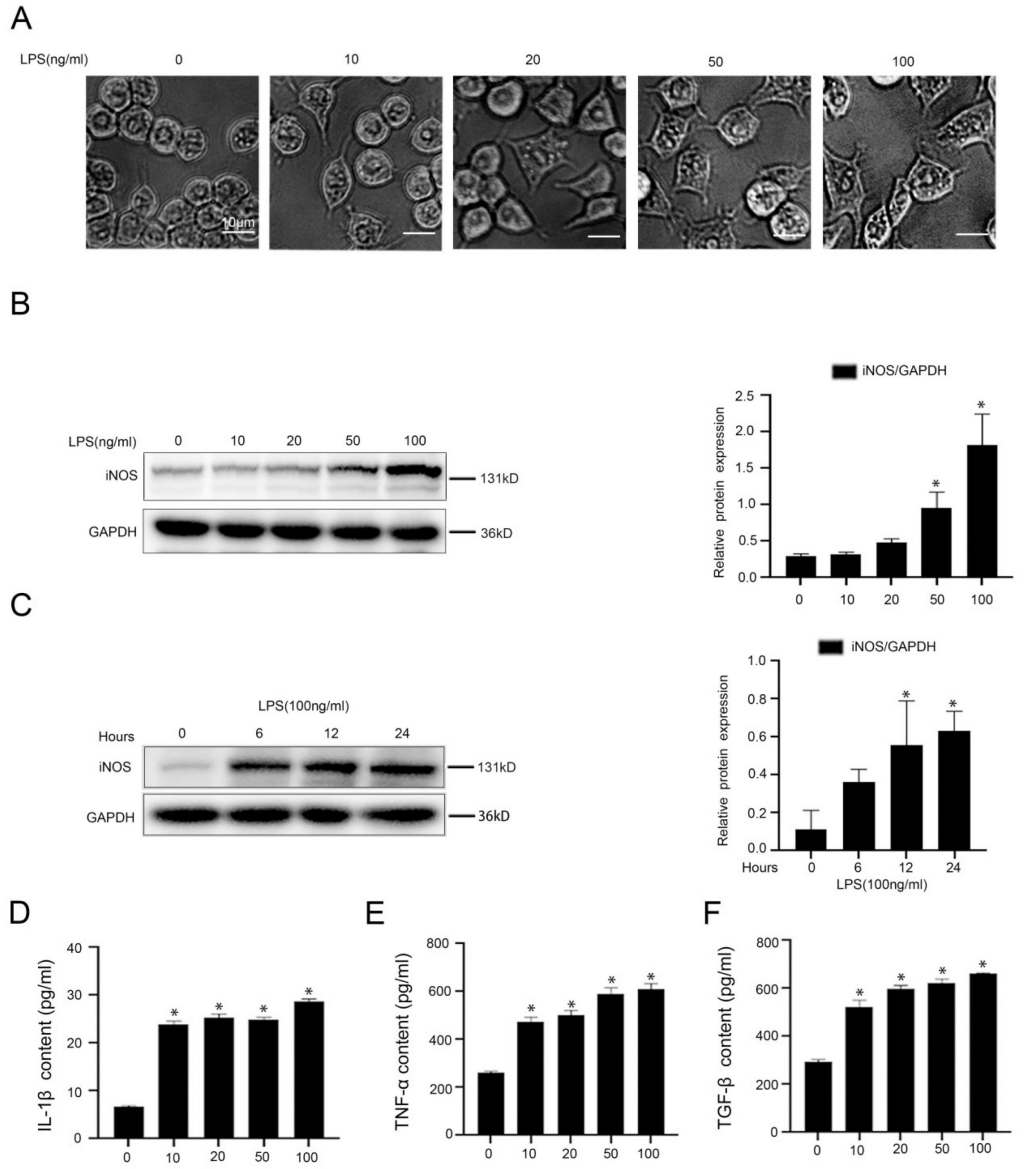
LPS induces the transformation of macrophages into pro-inflammatory M1 type. (A) Cell morphology of murine macrophages RAW 264.7 of the five groups (0, 10, 20, 50, 100 ng/ml), was visualized by light microscope (scale bar: 10 μm). (B) Representative immunoblots of iNOS of murine macrophages RAW 264.7 of the five groups (0, 10, 20, 50, 100 ng/ml). The relative protein expression was quantified in the graph, right. **(C)** Representative immunoblots of iNOS of murine macrophages RAW 264.7 of the four groups (0 h, 6 h, 12 h, 24 h with LPS (100 ng/ml)). The relative protein expression was quantified in the graph, right. (D-F) The content of IL-1β, TNF-α, and TGF-β in cell supernatant of the five groups (0, 10, 20, 50, 100 ng/ml). *P < 0.05 versus the 0 ng/ml group or the 0 h group.

To investigate the molecular mechanism of LPS-induced inflammation in macrophages, firstly, murine macrophages RAW 264.7 were stimulated with LPS at different doses (0, 10, 20, 50, 100 ng/ml) for 24hours or at the dose (100 ng/ml) from 0 hour to 24 hours *in vitro*. The light microscope displayed irregular morphology of macrophages, induced by LPS with the increase of concentration (Figure 3A). As we expected, there was a time-dependent and a concentration-dependent up-regulation of iNOS expression in LPS-treated macrophages (Figure 3B, C). Besides, the content of IL-1β, TNF-α, and TGF-β in the cell supernatant of LPS-treated macrophages was elevated, which was measured with ELISA (Figure 3D-F). These data suggested that LPS promoted the transformation of macrophages into pro-inflammatory M1 type and the secretion of inflammatory cytokines in macrophages.

**Figure 4:**
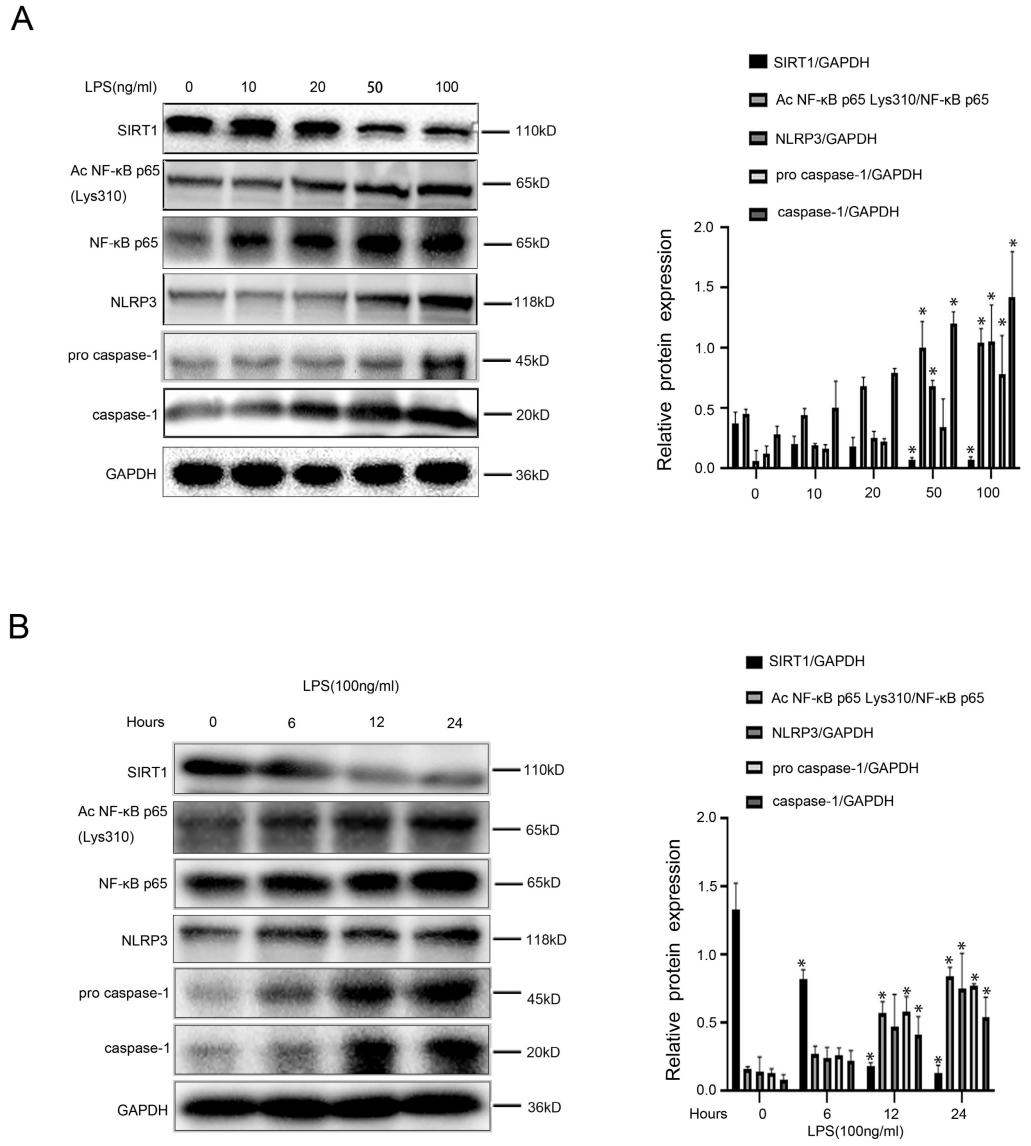
LPS-induced NF-KB p65 acetylation mediates activation of NLRP3. (A) Representative immunoblots of SIRT1, NF-κB p65, Ac NF-κB p65 Lys310, NLRP3, pro-caspase-1, and caspase-1 of murine macrophages RAW 264.7 of the five groups (0, 10, 20, 50,100 ng/ml). The relative protein expression was quantified in the graph, right. (B) Representative immunoblots of SIRT1, NF-κB p65, Ac NF-κB p65 Lys310, NLRP3, caspase-1 of murine macrophages RAW 264.7 of the four groups (0 h, 6 h, 12 h, 24 h with LPS (100 ng/ml)). The relative protein expression was quantified in the graph, right. *P < 0.05 versus the 0 ng/ml group or the 0 h group.

Intriguingly, we found that in LPS-treated macrophages, protein levels of Ac NF-κB p65 Lys310, NF-κB p65, NLRP3, pro-caspase-1, and caspase-1 increased with time and concentration; however, protein expression of SIRT1 was reduced by LPS (Figure 4A, B). These results indicated that LPS likely up-regulated acetylation of NF-κB p65, and subsequently activated the NF-κB/NLRP3 pathway, leading to the inflammation in macrophages.

**Figure 5:**
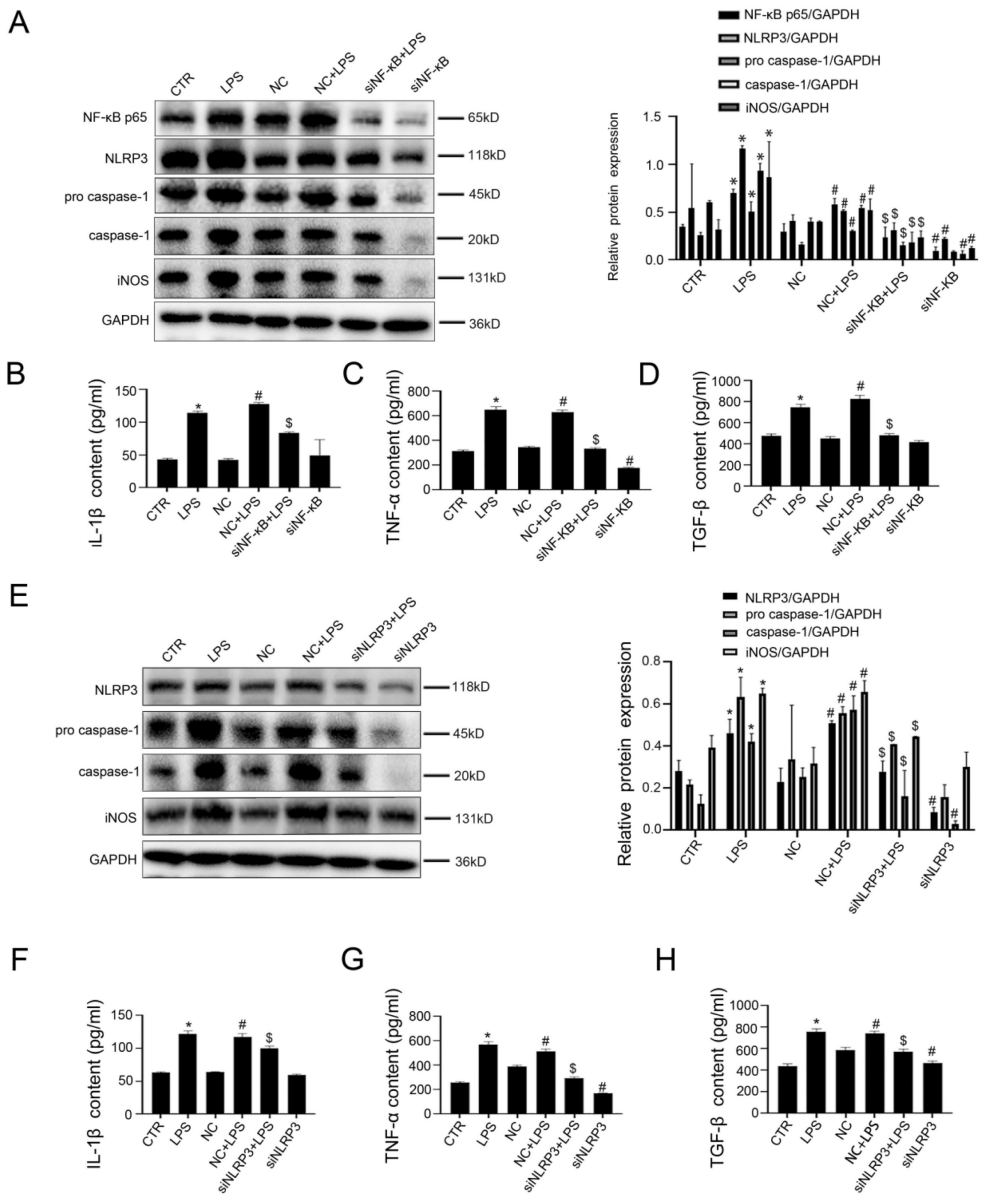
Inhibition of NF-κB and NLRP3 restrains the transformation of macrophages into pro-inflammatory M1 type. (A) Representative immunoblots of NF-KB p65, NLPR3, pro-caspase-1, and caspase-1 of murine macrophages RAW 264.7 of the six groups (CTR, LPS, NC, NC + LPS, si NF-κB+ LPS, and si NF-κB). The relative protein expression was quantified in the graph, right. (B-D) The content of IL-1β, TNF-α, and TGF-β in cell supernatant of the six groups (CTR, LPS, NC, NC + LPS, si NF-κB+ LPS, and si NF-κB). (E) Representative immunoblots of NLPR3, pro-caspase-1 and caspase-1 of murine macrophages RAW 264.7 of the six groups (CTR, LPS, NC, NC + LPS, siNLRP3+ LPS and siNLRP3). The relative protein expression was quantified in the graph, right. (F-H) The content of IL-1β, TNF-α, and TGF-β in cell supernatant of the six groups (CTR, LPS, NC, NC + LPS, siNLRP3+ LPS and siNLRP3). *P < 0.05 versus the CTR group; ^#^P < 0.05 versus the NC group; ^$^P < 0.05 versus the NC + LPS group.

Next, murine macrophages RAW 264.7 were transfected with NF-κB siRNA and NLRP3 siRNA or non-target siRNA (called NC), and then stimulated with LPS (100 ng/ ml) for 24 hours. The transfection efficiency reached 75%. As expected, the western blotting showed that, compared with the control group and the NC group, the protein expression of NF-κB p65, NLRP3, pro-caspase-1, caspase-1, and iNOS increased in the LPS group and the NC+LPS group; conversely, si NF-κB and si NLRP3 could inhibit these effects (Figure 5A, E). Moreover, LPS-induced upregulation of IL-1β, TNF-α, and TGF-β in cell supernatant was reduced by silencing NF-κB or NLRP3 with siRNA (Figure 5B-D, F-H).

Hence, these results suggested that LPS-mediated acetylation of NF-κB P65 induced activation of NLRP3, thereby promoting the transformation of macrophages into pro-inflammatory M1 type.

### Activating SIRT1 inhibits the inflammation in macrophages

**Figure 6:**
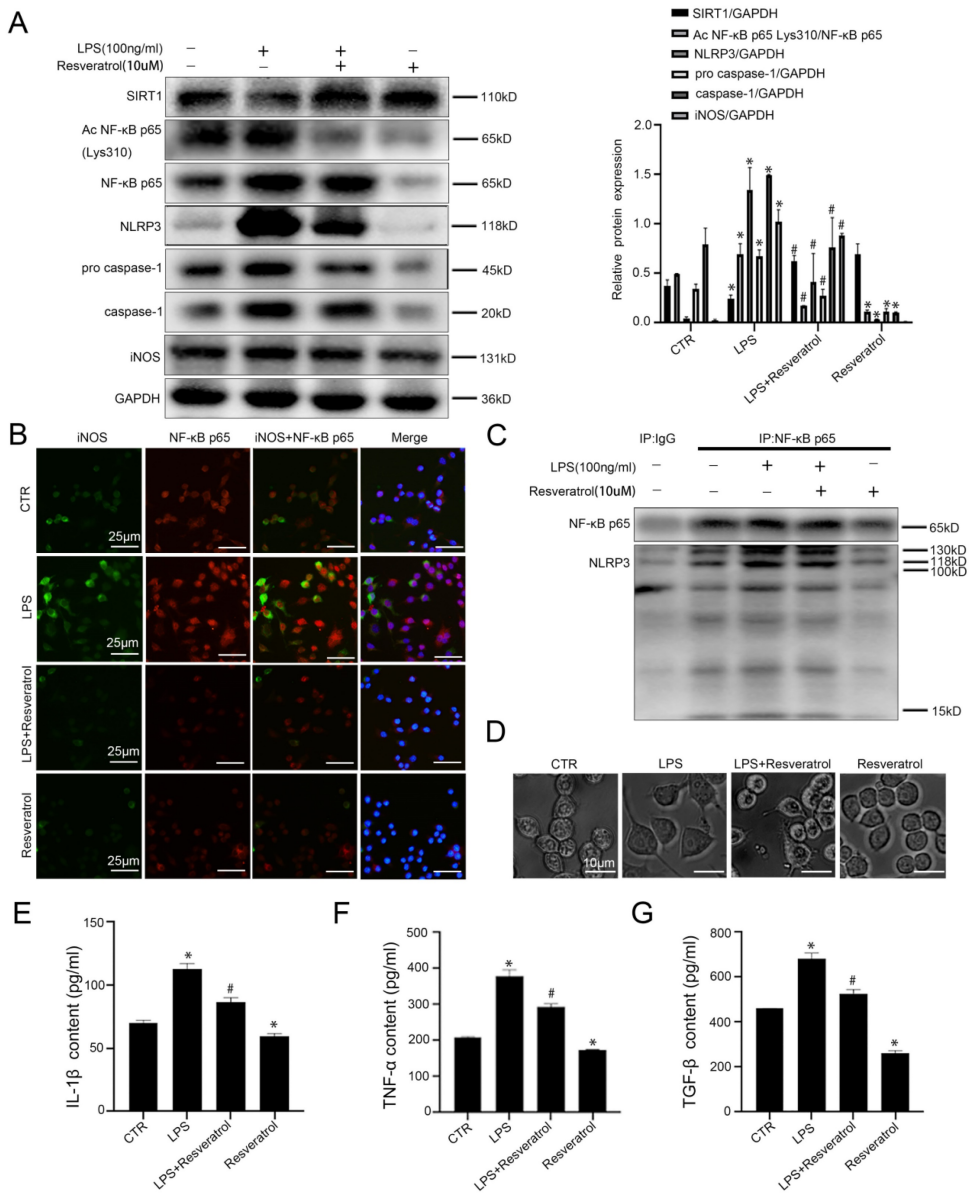
Activation of SIRT1 promotes deacetylation of NF-κB p65 which inhibits NLRP3 activation and the secretion of inflammatory cytokines in macrophages. (A) Representative immunoblots of SIRT1, Ac NF-κB p65 Lys310, NF-κB p65, NLRP3, pro-caspase-1, caspase-1, and iNOS of murine macrophages RAW 264.7 of the four groups (CTR, LPS, LPS+Resveratrol, Resveratrol). The relative protein expression was quantified in the graph, right. (B) The expression of iNOS (green) and NF-κB p65 (red) of murine macrophages RAW 264.7, visualized by fluorescence microscopy (scale bar: 25 μm). Nuclear was showed by DAPI (blue). (C) Interaction of NLPR3 with NF-KB P65 was detected by the co-IP assay. NF-κB p65 of RAW 264.7 was individually immunoprecipitated, as well as NF-κB p65 and NLRP3 subjected to immunoblotting analysis as indicated. (D) Cell morphology of murine macrophages RAW 264.7 of the four groups (CTR, LPS, LPS+Resveratrol, Resveratrol), was visualized by light microscope (scale bar: 10 μm). (E-G) The content of IL-1β, TNF-α, and TGF-β in cell supernatant of the four groups (CTR, LPS, LPS+Resveratrol, Resveratrol). *P < 0.05 versus the CTR group; ^#^P <0.05 versus the LPS group.

To investigate the molecular mechanism of SIRT1 in macrophages in liver inflammation and fibrosis, murine macrophages RAW 264.7 were pretreated with resveratrol (10 uM), and then stimulated with LPS (100ng/ml). Compared with the control group, the protein levels of Ac NF-κB p65 Lys310, NF-κB p65, NLRP3, pro-caspase-1, caspase-1, and iNOS increased significantly in the LPS group; however, activation of SIRT1 with resveratrol could inhibit their expression (Figure 6A). Besides, in agreement with these data, the immunofluorescence showed that more NF-κB p65 and iNOS expressed in LPS-treated murine macrophages RAW 264.7; whereas less expression of NF-κB p65 with iNOS was displayed in the LPS+resveratrol group and the resveratrol group (Figure 6B). Meanwhile, the co-immunoprecipitation (Co-IP) assay revealed that the co-precipitation of NF-κB p65 with NLRP3 was enhanced in the LPS group, whose interaction was disrupted by resveratrol (Figure 6C). The light microscope showed that, compared to the control group, cell morphology of macrophages in the LPS group became irregular; whereas these effects were reversed by activating SIRT1 with resveratrol (Figure 6D). In addition, resveratrol could attenuate LPS-induced high levels of IL-1β, TNF-α, and TGF-β in cell supernatant (Figure 6E-G).

**Figure 7:**
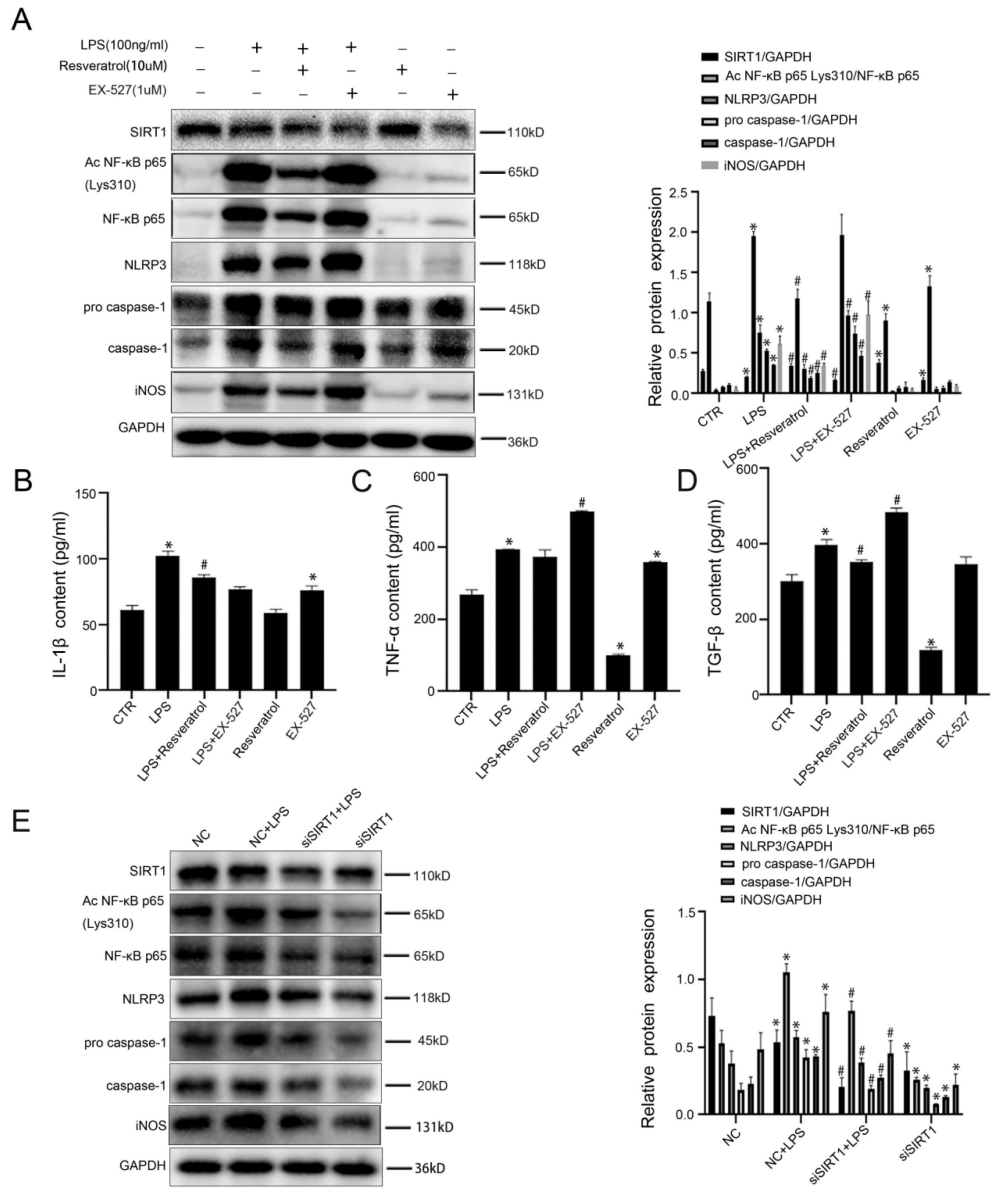
Inhibiting SIRT1 induces acetylation of NF-κB p65 which induces activation of NLRP3 and the secretion of inflammatory cytokines in macrophages. (A) Representative immunoblots of SIRT1, Ac NF-κB p65 Lys310, NF-κB p65, NLRP3, pro-caspase-1, caspase-1 and iNOS of murine macrophages RAW 264.7 of the six groups (CTR, LPS, LPS+Resveratrol, LPS+EX-527, Resveratrol, EX-527). The relative protein expression was quantified in the graph, right. (B-D) The content of IL-1β, TNF-α, and TGF-β in cell supernatant of the six groups (CTR, LPS, LPS+Resveratrol, LPS+EX-527, Resveratrol, EX-527). (E) Representative immunoblots of SIRT1, Ac NF-κB p65 Lys310, NF-κB p65, NLRP3 pro-caspase-1, caspase-1 and iNOS of murine macrophages RAW 264.7 of the four groups (NC, NC + LPS, si SIRT1+ LPS, and si SIRT1). The relative protein expression was quantified in the graph, right. *P < 0.05 versus the CTR group or the NC group; ^#^P < 0.05 versus the LPS group or the NC+LPS group.

Furthermore, to demonstrate that resveratrol can reduce inflammation in macrophages by up-regulating SIRT1 expression, murine macrophages RAW 264.7 were pretreated with resveratrol or EX-527 (a SIRT1 inhibitor), and then stimulated by LPS; meanwhile, murine macrophages RAW 264.7 were transfected with SIRT1 siRNA, and then stimulated with LPS (100 ng/ ml) for 24 hours, the transfection efficiency reached 75%. The western blotting showed that compared with the control group and the LPS group, the protein levels of Ac NF-κB p65 Lys310, NF-κB p65, NLRP3, pro-caspase-1, caspase-1, and iNOS were up-regulated in the LPS+EX-527 group, with the decrease of SIRT1 expression (Figure 7A). Meanwhile, EX-527 increased the high levels of IL-1β, TNF-α and TGF-β in the LPS-induced cell supernatant (Figure 7B-D). Besides, the western blotting showed that, compared with the NC group, the protein levels of Ac NF-κB p65 Lys310, NF-κB p65, NLRP3, pro-caspase-1, caspase-1, and iNOS were increased in the NC+LPS group; on the contrary, the expression of these proteins was decreased by silencing SIRT1 with siRNA (Figure 7E).

The results demonstrated that activation of SIRT1 inhibited the NF-κB/NLRP3 pathway to attenuate the inflammation in macrophages via deacetylation of NF-κB p65. However, inhibition or Knockdown of SIRT1 induced macrophages to transform into pro-inflammatory M1 type through acetylating NF-κB p65 and up-regulating the NF-κB/NLRP3 pathway.

### Inhibiting inflammation in macrophages via activating SIRT1 could reverse activation of HSCs

**Figure 8:**
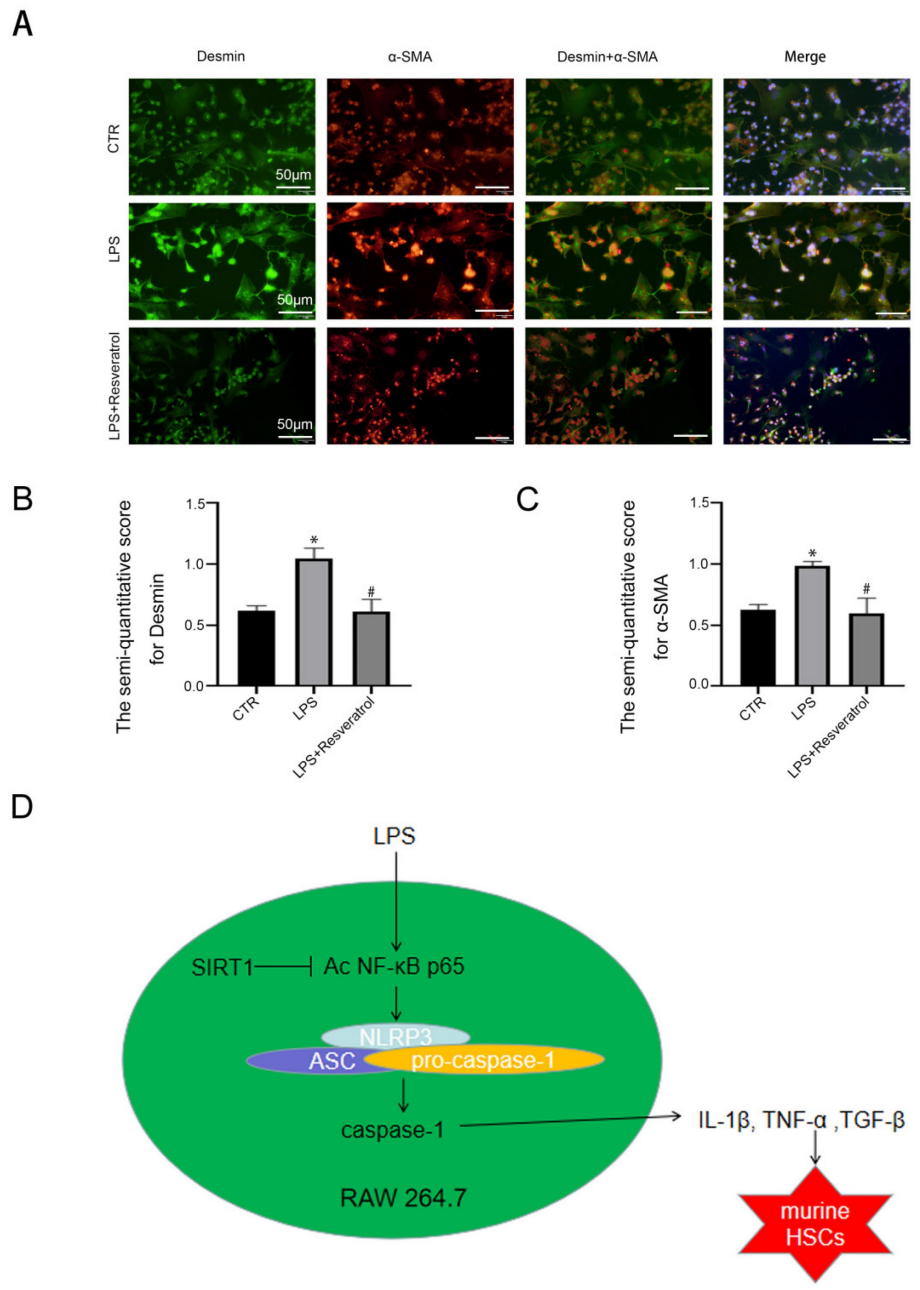
Hepatic macrophages release inflammatory cytokines to activate HSCs. (A) The α-SMA protein expression (red) and the Desmin protein expression (green) in murine HSCs of the three groups (CTR, LPS, LPS+Resveratrol), shown by immunofluorescence. Nuclear was showed by DAPI (blue) (scale bar: 50 μm). (B-C) The semiquantitative score of immunofluorescent for Desmin and α-SMA in the two graphs. *P < 0.05 versus the CTR group; ^#^P <0.05 versus the LPS group. (D) A schematic view of major signal transduction pathways, involves in the conclusion that sirt1 inhibits the transcriptional activity of NF-κB p65 by down-regulating its expression and acetylation level, further inhibiting the activation of NLRP3 and the release of inflammatory factors, thus inhibiting the activation of HSCs to alleviate liver inflammatory and fibrosis.

At last, to evaluate the interplay between regulating SIRT1 in macrophages and activation of HSCs, murine macrophages RAW 264.7 were pre-treated with LPS and resveratrol for 24 hours, and subsequently co-cultured with primary murine HSCs. Immunofluorescence displayed that the α-SMA expression was increased in the LPS group; in contrast, less α-SMA was expressed in the LPS + resveratrol group (Figure 8A-C). The observation suggested that HSCs were activated due to co-culture with LPS-treated macrophages; in contrast, activating SIRT1 with resveratrol in macrophages could reduce activation of HSCs.

Taken together, the results indicated that activating SIRT1 in macrophages could reverse activation of HSCs through relieving the inflammation of macrophages, thereby alleviating liver fibrosis.

## Discussion

Our present study discovers that LPS triggers the transformation of macrophages into pro-inflammatory M1 type and the release of inflammatory cytokines via acetylation of NF-κB p65, and subsequently activates HSCs; both activating SIRT1 with resveratrol and overexpressing SIRT1 with adenovirus vector promotes deacetylation of NF-κB p65, and then relieves inflammatory of macrophages by down-regulation of NF-κB/NLRP3 pathway and inhibits activation of HSCs, thereby alleviating hepatic inflammatory and fibrosis (Figure 8D).

In the absence of disease, the mucosal barrier in the gut remains intact, preventing the transportation of microorganisms from the intestinal lumen to the liver. However, in chronic liver disease (CLD), due to damage of the intestinal barrier, microorganisms and their products migrate into the bloodstream and circulate in the portal vein, leading to a strong inflammatory response in the liver 16. Studies have shown that the gut microbiome and its products, especially endotoxins produced by Gram-negative bacteria, are considered to be one of the major factors accelerating the progression of liver disease. Clinical studies have found that in patients with chronic liver disease, dysbiosis and intestinal mucosal barrier destruction are often observed, and plasma endotoxin level increases with the development of liver fibrosis stage 17, 18. Our study also found that the plasma LPS level increased in CCl4-treated rats, indicating that intestinal permeability and intestinal LPS leakage increased in the process of CCl4-induced liver fibrosis in rats. Therefore, in cell experiments, we used LPS to stimulate mouse macrophages RAW 264.7 to establish an inflammatory model, and further investigate the role of macrophages in liver inflammation and fibrosis.

Hepatic macrophages are considered to be the first line of defense against pathogens and are involved in all stages of hepatic fibrosis, from the onset of inflammation and progression of fibrosis 19. It is known that classically activated M1-type macrophages are involved in triggering and maintaining inflammation 20, while iNOS is a signature molecule of M1-type macrophages 21. Activation of hepatic macrophages induces secretion of pro-inflammatory cytokines that can drive the activation and proliferation of primary HSC and inhibit its apoptosis 2. As we know, hepatic stellate cells are considered to be the primary effector cells of liver fibrosis 22. In our study, we found that CCl4 induced circulating LPS and high expression of iNOS in CCl4-induced hepatic steatosis and fibrosis rat models. In addition, we similarly found that abnormal activation of macrophages occurred in LPS-treated RAW 264.7. These data indicated that liver inflammation and fibrosis may be related to the transformation of macrophages into the pro-inflammatory M1 type. However, the role of hepatic macrophages in liver inflammation and fibrosis is still elusive.

Next, we further explored the role of macrophages in inducing liver inflammation and fibrosis. NF-κB, an important inducible transcription factor, is a heterodimer composed of p50 and RelA/p65 peptides that controls the expression of many inflammatory related genes 23, 24. Acetylation of RelA/P65 at lysine 310 residues is critical for sustained release of pro-inflammatory cytokines and is also the key to fully activating the transcriptional potential of NF-κB 25, 26. NF-κB not only promotes transcription of cytokines, but also mediates activation of the NLRP3 inflammasome, which plays a key role in immune and inflammatory regulation. Activation of NLRP3 inflammasome transformed pro-caspase-1 into caspase-1, thereby promoting the secretion of inflammatory factors, such as IL-1β and IL-18 27. Recently, it's reported that abnormal activation of NLRP3 inflammasome may lead to the progression of liver disease in patients or mice 28. Our study displayed that during the progression of CCl4-induced hepatic steatosis and fibrosis, CCl4 induced the expression of NF-κB p65, NLRP3, iNOS, and α-SMA but inhibited the expression of SIRT1; *in vitro*, with the reduction of SIRT1 expression, NF-κB/NLRP3 pathway was upregulated, along with the high level of Ac NF-κB p65 Lys310 and the release of inflammatory cytokines in LPS-treated RAW 264.7; whereas knockdown of NF-κB or NLRP3 could rescue these effects. These data demonstrated that the release of inflammatory cytokines initiates liver inflammation and fibrosis by the up-regulation of NF-κB/NLRP3 signaling pathway through acetylating NF-κB p65 in macrophages.

SIRT1 is a NAD [+] dependent protein deacetylase that inhibits NF-κB signal transduction by deacetylating the P65 subunit of NF-κB, leading to a reduction in the inflammatory response mediated by this transcription factor 29, 30. Some recent evidence reports that the activation of SIRT1 confers protective effects on liver inflammation and fibrosis 13, 14. Whereas little research explores the role of SIRT1 in hepatic macrophages. At the same time, studies have shown that activation of SIRT1 can maintain intestinal epithelial barrier function, stabilize intestinal epithelial permeability, and reduce serum LPS level 31-32. *In vivo* studies showed that overexpression of SIRT1 on day 28 could inhibit the increase of plasma LPS induced by CCl4. We believe that this is related to alleviating liver fibrosis and reducing intestinal permeability by overexpression of SIRT1. However, overexpression of SIRT1 did not significantly reduce plasma LPS level on day 6, which may be related to the fact that the intestinal mucosal barrier was not significantly improved in a short period of time. *In vitro*, LPS activated acetylation of NF-κB p65 in RAW 264.7; meanwhile, more NF-κB p65 and iNOS were expressed in LPS-treated RAW 264.7. However, activating SIRT1 with resveratrol or overexpression of SIRT1 with adenovirus vector inhibited macrophages into pro-inflammatory M1 type and the release of inflammatory cytokines via downregulation of the NF-κB/NLRP3 pathway by deacetylating NF-κB p65. Meanwhile, activating SIRT1 with resveratrol inhibited the co-precipitation of NF-κB p65 with NLRP3 and activation of HSCs. Whereas inhibition or Knockdown of SIRT1 expression aggravated NLRP3-associated inflammation in macrophages. Therefore, activating SIRT1 alleviates liver inflammation and fibrosis through inhibiting the transformation of macrophages into pro-inflammatory M1 type and the activation of HSCs by deacetylating NF-κB p65.

Although this study provides new insights into the regulatory mechanism of SIRT1 in liver inflammation and fibrosis, our experiment still has some limitations. The role of macrophages in liver inflammation and fibrosis remains to be further investigated. It's also not completely clear how to exert anti-fibrosis effect by regulating phenotypic transformation of macrophages.

## Conclusions

In summary, we have established that the expression level of SIRT1 decreased in LPS-treated macrophages accompanied by the upregulation of acetylation level of NF-κB p65. Meanwhile, the results show that LPS promotes the inflammation of macrophages by up-regulating NF-κB/NLRP3 pathway, and then inducing the activation of HSCs. Activating SIRT1 deacetylated NF-κB p65 to alleviate liver inflammation and fibrosis via inhibiting the NF-κB/NLRP3 pathway in macrophages. This provides a new approach to explore novel therapeutic targets for hepatic fibrosis.

## Abbreviations

CCl4: carbon tetrachloride
HSC: hepatic stellate cell
iNOS: inducible nitric oxide synthase
IL-1β: interleukin-1β
IHC: immunohistochemistry
KCs: kupffer cells
LPS: lipopolysaccharide
MoMFs: monocyte derived macrophages
SIRT1: silencing regulatory protein 1
α-SMA: α-smooth muscle actin
